# Adaptation and Evaluation of the Neighborhood Environment Walkability Scale in India (NEWS-India)

**DOI:** 10.3390/ijerph13040401

**Published:** 2016-04-02

**Authors:** Deepti Adlakha, J. Aaron Hipp, Ross C. Brownson

**Affiliations:** 1 Center for Public Health, School of Medicine, Dentistry and Biomedical Sciences, Queens University-Belfast, Belfast BT7 1NN, UK; 2 Department of Parks, Recreation, and Tourism Management and Center for Geospatial Analytics, College of Natural Resources, North Carolina State University, Raleigh, NC 27695, USA; jahipp@ncsu.edu; 3 Prevention Research Center, Brown School, Division of Public Health Sciences and Siteman Cancer Center, School of Medicine, Washington University in St. Louis, St. Louis, MO 63130, USA; rbrownson@wustl.edu

**Keywords:** India, walkability, built environment, physical activity, measurement

## Abstract

Physical inactivity is the fourth leading risk factor for global mortality, with most of these deaths occurring in low and middle-income countries (LMICs) like India. Research from developed countries has consistently demonstrated associations between built environment features and physical activity levels of populations. The development of culturally sensitive and reliable measures of the built environment is a necessary first step for accurate analysis of environmental correlates of physical activity in LMICs. This study systematically adapted the Neighborhood Environment Walkability Scale (NEWS) for India and evaluated aspects of test-retest reliability of the adapted version among Indian adults. Cultural adaptation of the NEWS was conducted by Indian and international experts. Semi-structured interviews were conducted with local residents and key informants in the city of Chennai, India. At baseline, participants (*N* = 370; female = 47.2%) from Chennai completed the adapted NEWS-India surveys on perceived residential density, land use mix-diversity, land use mix-access, street connectivity, infrastructure and safety for walking and cycling, aesthetics, traffic safety, and safety from crime. NEWS-India was administered for a second time to consenting participants (*N* = 62; female = 53.2%) with a gap of 2–3 weeks between successive administrations. Qualitative findings demonstrated that built environment barriers and constraints to active commuting and physical activity behaviors intersected with social ecological systems. The adapted NEWS subscales had moderate to high test-retest reliability (ICC range 0.48–0.99). The NEWS-India demonstrated acceptable measurement properties among Indian adults and may be a useful tool for evaluation of built environment attributes in India. Further adaptation and evaluation in rural and suburban settings in India is essential to create a version that could be used throughout India.

## 1. Introduction

Non-communicable diseases (NCDs) like obesity, diabetes, and cardiovascular disease constitute a significant portion of the growing health burden across the world, of which the greatest increases are expected in low- and middle-income countries (LMICs) [1,2,3,4,5,6]. India, a LMIC with a population of 1.2 billion people and soon to be the world’s most populous country, is experiencing a NCD epidemic [7,8,9]. India has the largest diabetic population in the world, with 33 million in 2015, projected to reach 130 million by 2030 [10,11,12,13,14,15,16]. Cardiovascular disease is the leading cause of death in India, and its contribution to mortality is rising; deaths due to cardiovascular disease are projected to increase from 2.7 million in 2004 to 4.0 million in 2030 [9,17,18,19]. Morbid obesity (body mass index (BMI) > 30 kg/m^2^) currently affects 5% of Indians (approximately 61 million people) [20].

Physical inactivity is a leading risk factor for development of NCDs [21,22,23,24,25]. Calls to reduce global epidemics of NCDs by the United Nations and the World Health Organization have recommended increasing physical activity (PA) as a key strategy [13,17,26,27]. In 2011, the United Nations High-level Meeting on Non-Communicable Diseases identified increasing PA as one of five priority intervention areas to reduce the impact of NCDs, noting modification of the built environment (BE) to support PA as a key focus area [28]. In this context, research linking the BE with PA has increased rapidly in recent years [29,30,31,32] and is now an international priority [33,34,35].

Mounting research evidence suggests that the BE can facilitate or constrain PA [36,37,38,39,40,41,42,43,44,45,46,47,48]. However, studies examining PA and BE associations thus far have been primarily limited to Australasia, Europe, North America, and South America [49,50,51]. Findings from these studies may not generalize to other parts of the world, particularly in LMICs like India that are collectively home to 80% of the world’s population and are at higher risk for developing NCDs [21,25,52].

The BE in many LMICs is distinct in terms of development patterns and different from those in the developed countries. Rapid, unplanned, and unsustainable urban development are making LMICs key focal points for emerging environmental and health hazards. These hazards include the inter-related problems of urban poverty, road traffic fatalities, and air pollution [53]. In addition, increased urbanization, greater dependence on automobiles, along with diminishing open space for walking and leisure in cities is associated with sedentary lifestyles or increased time spent sitting [54,55,56,57]. These lifestyle modifications have led to marked shifts in energy imbalance that are spawning the rise of NCDs [46,58,59,60]. This sum of influences that the surroundings, opportunities, or prevailing conditions of life have on promoting obesity in individuals or populations has been termed obesogenic [61,62,63,64], and has not yet been fully understood in the context of LMICs.

Due to BE differences, questions remain about the applicability of surveys constructed in developed countries to the local contexts in LMICs. BE correlates of PA that have been documented in developed countries have yet to be studied among LMICs like India. To address this issue, there have been recent calls for investigators to collaborate on a regional basis to adapt BE measures that are tailored to the LMIC contexts [33,65]. The development and testing of reliable and culturally sensitive measures of BE attributes is a necessary first step for accurate analysis of environmental correlates of PA in low-and-middle-income countries.

The methodology used in this study is based on the recommendations of the International Physical activity and the Environment Network (IPEN; www.ipenproject.org), an organization that has established common methods and measures for worldwide research on PA and BE’s. The survey instrument was adapted from the Neighborhood Environment Walkability Scale (NEWS) that has been used widely by IPEN [66,67,68,69,70]. The NEWS is a self-reported survey instrument to assess BE characteristics relevant for PA. Subscales of NEWS assess perceived residential density, land use mix, street connectivity, walking/biking infrastructure, and traffic and crime safety. This scale was developed in the United States (U.S.) in 2002, and has been successfully implemented in LMIC countries in Asia (China [71], Japan [72]), Africa (Nigeria [65,73]) and South America (Brazil [74]) in recent years.

This study is the first of its kind to conduct a cultural adaptation and evaluation of a BE survey—NEWS—in India. The present study was conducted in an understudied region in Asia and has the potential to add to the international literature on the relevance and impact of healthy environments.

## 2. Methods

### 2.1. Study Setting and Sampling Procedures

This study recruited a diverse sample of participants from the metropolitan area (164.48 sq. miles) of the city of Chennai, India. Chennai is the capital city of the state of Tamil Nadu, a major commercial and industrial hub in southern India [75,76]. Chennai is the fourth most populous city (8.9 million residents) in India. Local residents (*N* = 14) and key informants (*N* = 7) were recruited using a purposive sampling technique across the Chennai metropolitan area. Purposive sampling has been more effective in the instrument development and adaptation stage and recommended by researchers who have conducted similar studies of this nature in LMIC contexts [65,68,77]. The principal investigator identified a small pool of local residents from formerly established relationships with neighborhood associations, resident welfare associations, and local contacts. These residents, through their social networks, suggested other residents who were interested in participating in the study. In order to ensure selection of a diverse sample, effort was made to recruit residents from different neighborhoods across the city.

Inclusion and exclusion criteria for local residents and key informants were based on IPEN protocol for NEWS adaptation and studies conducted in Nigeria [65], Brazil [78], and China [71]. Eligibility criteria for local residents included: (i) current residents of Chennai metropolitan area; (ii) residents of the Chennai metropolitan area for at least 6 months; (iii) 18–65 years of age; (iv) being able and willing to answer questions in English or Tamil, which is the official language in the study region; (v) not having any disability that prevented independent walking; and (vi) no visible signs of cognitive impairment.

Key informants for this study were chosen on the basis of their interest and related work in local city planning, transportation engineering, walkability, PA, and obesity or diabetes-related research. A multidisciplinary group of seven key informants including but not limited to local city planners, transportation engineers, park and recreation professionals, geographers, and public health scientists were developed from the PI’s established contacts. Eligibility criteria for local experts included: (i) current residents of Chennai metropolitan area; (ii) 18–65 years of age; (iii) ability to speak English or Tamil; (iv) interest in BE and public health issues. Exclusion criteria for participants were: (i) presence of a medical condition that interfered with the ability to walk; (ii) unsuitable appearance (drunkenness, drug addiction, illegal possession of weapons). Local residents and key informants were excluded from the interviewing sample if they were less than 18 years of age and/or did not consent to being interviewed or audio-recorded.

### 2.2. Data Collection and NEWS Adaptation

Semi-structured interviews were conducted with local residents and key informants between August 2013 and January 2014. A list of potential residents and key informants was developed using the above-stated eligibility criteria. From this list, participants were contacted either in-person, *via* telephone or email and asked about their interest and eligibility to participate. All participants that were contacted consented to being interviewed. A convenient time, date, and location for the interview was set for the consenting participants. Research information sheets and interview questions were emailed to participants two days prior to the scheduled interview. Each interview lasted 30–40 min and was audio-recorded. All study procedures were approved by Washington University’s Human Research Protection Office (IRB ID #201410052) and followed Institutional Review Board Guidelines.

Residents were asked questions on the perception of their neighborhood BE as well as attitudes, beliefs, and intentions for PA. Key informants were asked to provide input on the original NEWS questionnaire and to think about environmental factors that are important for both cities and villages in India. Key informants were also instructed to help identify items on the NEWS that are not relevant to local environments in India and to suggest culturally appropriate and equivalent items in the Indian context. The goal of this adaptation process was to retain as many original concepts and items as possible, but to express them in ways that are appropriate for the local culture and environment. Most importantly, key informants were asked to suggest inputs on environmental factors that are important for PA in India that are missing on the original NEWS. Key informants were asked to consider both physical BE and social environment. Factors in the social environment included presence of violence, crime, gang activity, and indicators of social disorder such as graffiti, trash, and people begging.

This qualitative research phase of the study employed a grounded theory approach to support the sample size [79]. Grounded theory uses theoretical saturation of data as a guideline to determine the number of participants required for a detailed analysis [80]. Saturation has been defined as data satisfaction achieved when a researcher reaches a point where no new information is obtained from the collection of further data [80,81]. In this study, a situation of theoretical saturation was attained where no new categories or properties emerged from the gathering of further data. There are no fixed sizes or standard tests that can determine the required data for reaching saturation. However, based on previous key informant research, 10–15 interviews were expected to provide adequate in-depth information and saturation on emergent themes and issues [82,83]. Data from interviews were analyzed and a summary report of findings was sent to all participants post analysis.

Emerging themes from the interviews (Table 1) were discussed with an international panel of IPEN experts including developers of the original NEWS-Adult (Sallis, Cain, Geremia and Conway at the University of California, San Diego, and Reis at Pontifícia Universidade Católica do Paraná, Curitiba, Brazil). These international experts ensured that the underlying concepts assessed by the NEWS questionnaire were not compromised during the adaptation process. Feedback from this panel was used to inform the adaptation of the NEWS-India. The final consensus version of NEWS-India included all items of the original instrument with their original wording or slightly modified form [69,70], with 24 additional items describing features of the environment relevant to India. The adapted NEWS-India consists of 91 items that assessed the following perceived environmental characteristics: (a) residential density (seven items); (b) land use mix-diversity (43 items); (c) land use mix-access (seven items); (d) street connectivity (five items); (e) infrastructure and safety for walking and bicycling (13 items); (f) aesthetics (six items); (g) traffic safety (six items); and (h) safety from crime (four items).

The adapted NEWS-India developed in this step was translated into Tamil, the official language of the state of Tamil Nadu and predominantly spoken in the study region. A knowledgeable bilingual person conducted the translation using terms and concepts that were understood by people residing in Chennai. These translations were reviewed by a group of bilingual people that are similar to the intended users, *i.e.*, residents of Chennai from a wide range of education levels and income groups. The group ensured that the Tamil translation of NEWS was acceptable to monolingual people. Two bilingual persons who were not familiar with the project and representative of eventual study participants (e.g., low socio-economic status) translated the new Tamil version of NEWS-India back into English (back translation). The back-translation was not required to produce the exact original wording. A group of bilingual people reviewed the back-translation and decided on the final translated version of NEWS-India. This process ensured that the meanings of the two versions were comparable. To assess the comparability of item wording, response options, and number of items, the study investigator provided back translations of surveys to two independent raters who were experts in the area (James F. Sallis, University of California, San Diego, CA, USA; Rodrigo Reis, Pontifícia Universidade Católica do Paraná, Curitiba, Brazil).

### 2.3. Pilot Testing and Cognitive Response Testing

NEWS-India was interviewer administered to 10 consenting adults for cognitive response testing. Cognitive response testing or cognitive interviewing is routinely used to refine questionnaires to enhance the quality of data collected [84,85]. To encourage critical feedback of the NEWS-India, participants were informed that the questionnaire was originally developed in the U.S. to assess attributes of the neighborhood BE that are important for PA in developed countries. Participants were interviewed separately and/or in a focus group, for their understanding of the words in the questionnaire, clarity of each item, and their suggestions for improvement. Participants were asked to verbalize their process of: (i) question comprehension (clarity of words, terms); (ii) information retrieval (response recall time); and (iii) decision making (aspects considered when choosing the response). Participants were also asked if any questions made them feel uncomfortable and if any relevant item in the local context was not included in the questionnaire. All participants were encouraged to verbalize their thought process while providing responses to the items. Results from this pilot test were discussed with the international expert panel and subsequently used to modify NEWS-India.

### 2.4. Survey Administration and Psychometric Testing

Between December 2014 and May 2015, NEWS-India was interviewer administered to consenting participants (*N* = 370). This phase of the study adopted a stratified two-stage cluster sampling strategy. Study participants were selected from neighborhoods chosen to maximize the variance in neighborhood walkability and socio-economic status (SES). This type of stratification by SES was used to enhance the representativeness of the sample because low-SES populations tend to be underrepresented in studies of this nature [67,86]. The goal of this phase of the study was to select participants from wards stratified into four quadrants that represent the following criteria: high-walkable/high-SES, high-walkable/low-SES, low-walkable/high-SES, and low-walkable/low-SES. To stratify neighborhoods by SES, IPEN studies have used median household income obtained from appropriate government ministries, departments or agencies [65,68]. Due to the lack of ward-level GIS and household income data for the city of Chennai, Walk Score was used to classify wards based on walkability and cost of rental units per square foot to define ward-level SES. Walk Score is a large-scale, public access walkability index that assigns a numerical walkability score to any address using a patented system [87]. A score above 90 (maximum 100) is a “Walkers Paradise” whereas below 24 is “Car Dependent”. Scores are calculated based on proximity to nearby amenities such as parks, groceries, schools, and public transit. Points are awarded based on the walking distance to amenities using distance decay calculations [87,88]. For example, amenities within a 5 min walk (0.25 miles) are given maximum points and more distant amenities receive minimum points, with no points given after a 30 min walk [89]. Walk Score also uses population density and road metrics such as block length and intersection density are used to measures pedestrian friendliness of a neighborhood [87,89].

Neighborhoods (wards) were divided into ten equal groups (deciles) based on their walkability and SES levels. Participants were recruited from identified neighborhoods using a purposive sampling technique and eligibility criteria discussed in Section 2.1. NEWS-India was administered for a second time to consenting participants (*N* = 62) with a gap of 2–3 weeks between successive administrations. Participants were contacted either in-person, via telephone or email, with up to 6 contact attempts to assess study interest and eligibility.

## 3. Data Analysis

### 3.1. Qualitative Analysis

Inductive and thematic analysis was conducted across the interview transcripts, using a framework approach to classify data according to key themes and emergent categories [90]. Data analysis included creation of nodes for certain themes and topics called coding. A node is like a container for qualitative data (themes, concepts) and presents a way to tag data in order to find and analyze trends or patterns [91]. A node hierarchy was created as a way of organizing nodes into main themes (parent nodes) and sub-themes (child nodes). Two research members read each transcript and identified coding themes and nodes. Themes were recorded and shared with the research team to develop a consistent coding scheme to be used within NVivo 10 (QSR International, Burlington, MA, USA) [92,93,94]. One team member present at the interview and one not present then coded each transcript using the developed coding scheme.

There were five broad categories of original interest, as structured in the semi-structured interview questions: neighborhood environment characteristics including pedestrian infrastructure, patterns of commuting, constraints to walking/bicycling and other types of PA, desired changes in infrastructure for the benefit of physical, psychological, and social well-being, and role of city-community partnerships in neighborhood planning and maintenance. As the nodes for coding the data were identified, several distinct patterns were recognized. Constant comparison was used to further investigate these patterns across social ecological systems in a matrix form similar to that used by Zayas *et al.* [94]. Socio-ecological systems refer to people’s interactions with their physical and sociocultural surroundings [36,95]. Ecological models of health behavior predict the most effective interventions should work on multiple levels to change psychological, social, policy, and physical environmental factors [36,96]. Numerous authors [97,98,99,100,101] and authoritative groups have identified environmental and policy interventions as the most promising strategies for creating population-wide improvements in eating, physical activity, and obesity, including the World Health Organization [13], Institute of Medicine [102,103], International Obesity Task Force [104] and Centers for Disease Control and Prevention [105,106]. However, environmental and policy factors are the least-studied category of physical activity correlates [107,108].

### 3.2. Assessment of Reliability

The reliability of the items of the adapted NEWS-India was assessed in two ways: the agreement of scores using the calculation of the kappa statistic for each item and one-way model single-measure intraclass correlation coefficients (ICC). Portney and Watkins suggest that when the unit of measurement is on a categorical scale, reliability can be assessed using a measure of agreement [109]. A simple index of agreement is the proportion of occasions that raters agree on scores, although this measure is limited as it does not take into account the level of agreement that could have occurred by chance. The kappa statistic overcomes this limitation by providing a chance-corrected measure of agreement.

To evaluate the test-retest reliability of the adapted NEWS-India, one-way model single-measure intraclass correlation coefficients (ICC) were calculated to test individual items. To test NEWS scale scores computed from multiple items, the single-measure ICC was also computed. ICC represents the proportion of total variance in a set of values that is attributable to between-subjects variability, with the remaining proportion attributable to error. ICC estimates >0.75 were considered as good reliability scores, between 0.50 and 0.75 as moderate reliability and <0.50 as poor reliability [110]. Statistical tests were conducted using the Statistical Package for the Social Sciences (SPSS) version 22 (IBM Corporation, Armonk, NY, USA) [111].

## 4. Results

In the process of performing the inductive analysis, it became salient that BE barriers and constraints to active commuting and PA behaviors were not only perceived by individuals, but constraints also intersected with socio-ecological systems. Though some specific constraints were unique to the individual and to the specific neighborhoods, there were mostly shared constraints. In total, themes addressed in the interviews were organized into the social ecological categories: micro, meso, exo, and macro-level factors [83,112]. Micro-level factors included perceived constraints at the individual level (e.g., overcrowding, lack of maintenance and cleanliness). Meso-level factors influenced PA behaviors in interpersonal specific user groups (e.g., women, older adults) across behavioral settings (e.g., poor pedestrian infrastructure and public transport access, lack of safety from traffic). Exo-level factors equally constrained PA participation by all members of the community (e.g., crime, gender-based violence, loss of sense of community) and macro factors were society-level constraints (e.g., disordered city planning, absence of road safety policies and law enforcement). Examples of socio-ecological factors at these levels are presented in Table 1.

### 4.1. Micro

Several perceived individual-level factors were identified. Residents cited proximity to work/school and access to diverse destinations as reasons for neighborhood selection. Micro-level constraints were identified as limiting individuals from being physically active and were a common narrative acknowledged in several interviews with residents. For example, lack of maintenance of neighborhood parks and playgrounds constrained the use of these spaces for PA among multiple participants. Individuals spoke of specific barriers and instances of constraints to PA due to lapses in maintenance and cleanliness in their neighborhoods. Overcrowding, disorderly traffic, and lack of sidewalks were identified as barriers to PA. For example, in reference to sidewalks with gaps and in disrepair, residents (R) said;

*R8:*  “Pavements are dirty, it is not at all good for walking.”

*R10:*  “For the pedestrians there is no space in the city. There are no proper pavements in most parts of the city. If there are pavements also, the bikes (motorized) will travel on the pavements. There is no respect for pedestrians in the city.”

*R10:*  “I do walk, but it’s not a very good experience. The pavements will be dug up and you won’t have any place to walk on them. It’s such a narrow road and you have buses coming. I don’t mind buses but it’s crazy the way they drive, so I am really scared to walk.”

### 4.2. Meso

Perceived constraints to PA specific to user groups and between behavioral settings were identified. Residents reported rapid development and construction of apartment complexes and increased commercial activity in residential areas, resulting in loss of green cover. Scarcity of road space and insufficient parking spots to accommodate increases in motor vehicle ownership across households were discussed. Lack of pedestrian infrastructure to support walking was highlighted. Several residents expressed concerns about threats to safety from traffic, particularly for women and older adults in their families. One resident said:

*R3:*  “There is practically no sidewalk in any part of the city. People are seen walking on roads. Except for the time I go to the park in the morning, I have to walk on roads. Walking in the park is much safer in the sense you know where you are walking. Wherever there are sidewalks, they are not worth walking on. And the government has not given importance to sidewalks and cycling. Women and elderly find it difficult.”

*R12:*  “They (city government) have dug up the road and the pedestrians, they can’t walk. And my dad, his eye sight is very bad, so he is not allowed to go outside after dark because he cannot cross the road. Most of the accidents occur with cyclists and pedestrians. They are very prone to accidents because there is no security.”

Residents discussed the desire to engage in everyday PA, but attributed the inability to do so due to non-existent sidewalks, high volumes of unregulated vehicular traffic, and poor enforcement of traffic rules. A few long-term residents recalled walking for errands (e.g., to the grocery store, library, *etc*.) and engaging in outdoor recreation or leisure PA in local parks and playgrounds in previous years, but reported being increasingly inactive or sedentary for leisure at present (watching TV, playing video games, sleeping, doing household chores, sitting at a desk, *etc*.). Reasons for this were cited as overcrowding and lack of existing opportunities or places for outdoor leisure-time PA. Residents said:

*R2:*  “Earlier I would go out and walk, I mean, at least three to four times a week to run small errands, to go to the library, to go shopping, but that is not possible at all because the road is very bad due to the metro (metro rail construction) going on. The main road is one-way (one-way traffic) and the traffic volume is too much. It’s very difficult to cross the road.

*R1:*  “I used to bicycle to the beach every weekend. But nowadays, because the traffic is so dangerous, I have stopped using my bicycle. I spend my weekends watching TV and playing games on the computer. Sometimes I play volleyball with my friends on the beach.”

Older adults reported going to neighborhood parks the most. However, despite living in streets adjacent to a park, they reported being unable to walk to the park due to increased road traffic volumes. They most commonly drove to the park and then engaged in PA within the park boundaries.

*R2:*  “If you want to have a nice walk, there is a park but getting to the park is a 20-min walk and then you have to brave all the traffic and go to the park. So if the conditions were better I would not mind walking to the park and then having a walk in the park.”

### 4.3. Exo

Study participants mentioned a strong sense of community and presence of religious institutions as key reasons for neighborhood selection. One of the participants stated that the primary reason for buying their house was because their neighborhood had a high concentration of people from the ethnic group/Indian state they belonged to:

*R6:*  “One of the reasons they (parents) bought it (their house) may be like a sense of community because, you know, there are lot of demographics living in Chennai, and they were Malayalees and they had come from Kerala (southern Indian state). So it sort of grew up like a little “Mallu” (colloquial term for Malayalee-a type of ethnic group in India) colony. All the people around you were sort of neighbours, were Malayalees. Because of that a temple came up. The temple was originally a Kerala temple.”

Another participant mentioned that easy access to temples around their home was a key factor for choosing to live in their neighbourhood:

*R9:*  “My parents are extremely spiritual people and there are lots of temples around, so they like to go there.”

Several Participants also identified distinct community-level constraints that were consistent across different areas of Chennai where they lived. For example, one of the participants mentioned a lack of any face-to-face contact and feeling disconnected from the neighbors on the street. This has resulted in a loss in the sense of community:

*R2:*  “This used to be a friendly, clean, and quiet neighbourhood. Before we used to walk on the road, stand at least near the gate and wave at each other. Now we don’t have any communication with our neighbours.”

Participants discussed safety concerns and gender-based violence against women and girls in their neighborhoods, streets, and public spaces. Some female participants mentioned having inferior access to public transport, feeling unsafe when walking alone after dark, being subject to pointed stares, inappropriate comments, and sexual harassment. One of the participants narrated incidents of women being subject to inappropriate comments while walking on the streets:

*R14:*  “You can’t say crime but there are people standing, passing lewd comments.”

The prevalence of these forms of gender-based violence in the exo-level system has resulted in making the public space a restricted area for women and girls, eliminating freedom and the human right to participate in the cultural and social life of the community. Research on women using public spaces in Indian cities has found correlations with environmental design features and attitudes of society [113,114]. Many women in India are concerned about using public spaces alone due to fear of crime. A study in Chennai found that women using public spaces like parks confined their activities to just walking, and they only used spaces that they trust [114]. The study also showed that visitors to the beach included couples, families, groups of friends, and single men, but very rarely single women [114]. Studies have found that the design of the BE can make spaces inviting for women and discourage situations where women get harassed. For example, a public space that is busy, surrounded by shops and stores with movement of people, open on all sides with good lighting makes women feel safer [113,115].

### 4.4. Macro

At the macro or societal level, participants discussed attitudes and ideologies of the government and community organizations. Residents mentioned that roads were designed to be automobile-dependent, neglecting pedestrians and bicyclists. Key informants were critical of the government policies on transport and pedestrian infrastructure. A common theme across all key informants was government policies were one-sided by favoring the automobile over pedestrians and bicyclists, resulting in high pedestrian fatalities. Key informants said:

*KI1:*  “Till the government doesn’t prioritize that pedestrian infrastructure is important and think laterally, road safety will be completely neglected in our city.”

*KI5:*  “It (road safety) is a very practical difficult issue to go around because the traffic on the road in my opinion in India is a million times worse than any other city in the world. In many ways Indian traffic situation is really, really bad, not only because it is overly congested compared to any other city around the world but it is also multi-vehicle, from bikes to say, any concept you try to apply, there is always a loophole. For example, it is very difficult to simulate these things anymore because behaviour of a bike or “thela gaadi” (hand-drawn cart for transporting goods) and so on, is very different from the behaviour of a car. Bikes squeeze in, about 50%–60% of Chennai traffic are bikes, so the way they behave, is completely different from your typical discussion on traffic in say, America, or any other country in the West.”

Participants criticized the haphazard and uncoordinated nature of work across government departments. Residents spoke about a lack of political will among political leaders and no vision when it comes to planning for the future. Several residents of the city believed that decisions taken by local municipal leaders without giving much thought to future prospects were responsible for the haphazard growth in the city. Discussing this lack of foresight, a resident said:

*R5:*  “Building a city is no joke. It is not like “Lego” that you just, you know, put in a few pieces and “Oh the city is there”. You need proper foresight.”

Several participants discussed the need for greater collaboration among city departments and improved partnerships between the city and neighborhood associations for improvement and maintenance of infrastructure, roads, and pedestrian facilities.

*KI5:*  “All the things we just mentioned requires multiple departments and multiple coordination and multiple capacity.”

*R2:*  “If the government, the corporation, if they do things at that (community) level I'm sure the neighborhood will also join hands and see to it that their streets are encroachment free and they'll see to it that they're kept neat and clean. There are neighborhoods where they do have competitions and like the people on their own beautify their sidewalks and they give out prizes for all these things. So these are all the things people can do at the community level. Once they find that things are kept neat and clean, I’m sure they will do their part of it.”

### 4.5. Test-Retest Reliability

Table 2, Table 3, Table 4, Table 5 and Table 6 show the response frequency and mean score of each item on the first assessment of the adapted NEWS-India and its test-retest reliability scores. The ICCs of the sum scores of each of the eight subscales (residential density, land use mix diversity, land use mix access, street connectivity, infrastructure for walking/bicycling, aesthetics, traffic safety, and safety from crime) ranged from 0.85 to 0.98. All subscale ICCs were higher than 0.75, indicating excellent reliability. ICCs of the individual items ranged from 0.48 to 1.00 with the lowest scores for a particular item of the “infrastructure and safety for walking and bicycling” subscale. 

In total, reliability of 80 items was in almost perfect agreement, reliability of nine items was substantial and reliability of two items was moderate [110]. The moderate reliability scores were probably due to a lack of variance in the answers, as the proportion of agreement for the two items with moderate reliability was generally high (>0.70 for the two items with moderate ICC reliability).

## 5. Discussion

This study evaluated the reliability of the adapted NEWS measure in India. The findings indicated moderate to high test-retest reliability for most subscales of NEWS-India. There are no previously published reports of the reliability of existing BE instruments for Indian cities. To our knowledge, there has been no measure developed to estimate neighborhood BE variables and walkability in India so far. This is one of the first studies to undertake cultural adaptation and reliability testing of a neighborhood walkability scale in India. As this appears to be the first published description of the development of a comprehensive instrument designed to measure factors in the physical environment that may influence walking and bicycling in neighborhoods in India, there are several lessons to be learned from this study.

The qualitative component of this study sheds light on the perceptions of BE and neighborhood walkability in an urban community in India. Residents and key-informants in Chennai city highlighted several constraints in their neighborhood environment that hindered active commuting and PA behaviors. Constraints intersected with social ecological systems at micro, meso, exo, and macro levels. Though some specific constraints were unique to the individual and to the specific neighborhoods, there were mostly shared constraints.

Constraints perceived at the individual level (micro) included barriers that participants saw limiting their own specific use of their neighborhood BE. In this study most commonly perceived constraints were safety from traffic, safety from crime, lack of maintenance, and poor quality of pedestrian infrastructure. Micro-system or individual-level results confirm constraints identified in previous IPEN studies using NEWS that focused only on this level, including the identification of neighborhood safety and availability of facilities such as sidewalks and walking paths as key criteria for PA promotion [65,66,71,116]. Neighborhoods cannot afford every BE feature or attribute desired. However, some core features should be present for individuals to utilize. These include sidewalks or walking paths that are maintained and kept clean. While the individual level constraints expressed in this study served to reinforce previous research on BE and walking behaviors, the other levels of socio-ecological constraints added new contextual information.

Rapid urbanization, increase in motorized traffic, loss of trees, parks, and green spaces were identified as key limitations to outdoor walking, particularly among specific user groups (e.g., women, older adults). Research evidence indicates physical inactivity is generally more prevalent among girls and women than their male counterparts and older adults are less likely to meet recommended levels of PA [32,34,117,118,119,120,121]. Studies have also shown that women and older adults more frequently report unsuitable environments as barriers to PA [119,121,122]. The perceived barriers faced by women are likely to differ from those faced by other groups, such as by men [123]. Aging populations with mobility disabilities are likely more vulnerable to environmental constraints such as problems with narrow sidewalks, lack of curb ramps or street crossings, poor lighting, puddles, and poor drainage [31]. This is consistent with findings from this study where women and older adults cited poor pedestrian infrastructure as a deterrent to walking outdoors. Older adults were the most frequent users of neighborhood parks in this study, which has also been observed in studies from LMICs [121,124,125]. Older adults reported being unable to walk to the park due to lack of sidewalks and crosswalks. Studies have shown that public parks have an important role to play in facilitating PA [41,126]. They provide places for individuals to walk or jog, and many have specific facilities for sports, exercise, and other vigorous activities [127]. In order to access these parks, pedestrian infrastructure linking residential streets to neighborhood parks and green spaces can play a key role in promoting safe walking routes for women and aging neighborhood residents [41].

In the exo-level system, factors such as feelings of social exclusion and lack of social connectedness indicated underlying disparities. The level of social cohesion is a key cultural component of neighbourhoods that has the potential to reinforce existing health inequalities through shaping of BE amenities such as differentiated access to greenspace between subgroups of the local population. Several studies have shown a positive relationship between local greenspace availability, health of residents, and social cohesion [128,129]. Neighborhoods play a role in supporting healthy behavior via social and physical BE features. Seaman and Jones (2010) demonstrated that the idea of walkability includes perceptions of social cohesion at a community level and the level of felt integration and inclusion by individuals in their communities. Individual's feelings of integration and inclusion can potentially mitigate the effects of experiential barriers in the neighbourhood BE, such as evidence of anti-social behaviour and gender-based violence as mentioned by participants in this study. Improving access to parks, green spaces for all in urban communities involves more than providing high quality resources such as sidewalks, crosswalks, lighting, *etc.* Physical availability interacts with community contexts already established and a holistic understanding of BE barriers and facilitators to PA is required. Overall, findings from qualitative interviews underscored the importance of improving the neighborhood walkability in Chennai to promote PA engagement and active lifestyles.

The test-retest reliability scores of the items in NEWS-India (ICC = 0.48 to 0.99) were generally high with almost perfect strength of agreement, indicating that the items are generally reliable. The values reported for the NEWS subscales in the present Indian study are comparable to those reported for the original version in the USA (ICC = 0.58 to 0.80) [70], the Australian version (ICC = 0.62 to 0.88) [130], the Chinese translation of the abbreviated version (ICC = 0.57 to 0.99) [116], and the African version (ICC = 0.59 to 0.91) [65].

While the overall strength of agreement across items was high, some items assessing subjective qualities of the BE such as general levels of attractiveness (e.g., attractive natural sights, attractive buildings) and difficulty for PA (e.g., traffic makes it difficult to walk) had lower ICC’s in the substantial agreement range. This could be due to participants experiencing difficulty in subjectively assessing the items measuring attractiveness and difficulty for PA. Since these items were based entirely on subjective overall impressions, it could be expected that the scores for them would vary based on the participant’s previous experiences of walking and cycling. It is possible that these NEWS items that pertain to aesthetics and pleasantness of the environments are ubiquitously unstable, regardless of developmental pattern and differences across sampled neighborhoods. Perhaps there are ambiguities in what contributes to an attractive or pleasant environment, as well as their qualities, which contribute to lower reliability of these items.

Only two items—presence of bicycling facilities and walking paths connecting dead-end streets—had low ICCs (0.48, 0.50) indicating moderate agreement. These may be because these items were assessing rare BE features that did not exist in the study area. Reliability studies from LMICs such as Hong Kong [71,131], Brazil [74], and Nigeria [65] have also demonstrated moderate agreement of ICCs for the same items assessing pedestrian and bicycling infrastructure since they are not common features of neighborhoods in those countries. Although the uncommon items may be omitted when using the adapted NEWS in India, it is recommended to retain these items as part of the Indian version because items like bicycling facilities can become indicators of progress that would be difficult to measure in future if these items were deleted. Retaining these items is also essential for comparing these attributes across countries. The little variability observed in the responses to these items from this sample likely accounted for the low reliability for the items. Nevertheless, the majority of the items on the Indian version of NEWS, including newly developed items, demonstrated moderate to almost perfect agreement in reliability coefficients, comparable to those found in other studies [70,73,116,130,132].

While these results indicate that the items in the NEWS-India instrument are generally reliable, this study has some limitations. First, conducting the study among residents of four neighborhood types from only one city in India may restrict environmental variability. Restricted variability could underestimate the strengths of BE-PA associations in environmental studies [35,133]. There was limited variation among the neighborhood quadrants assessed and the number of participants that were assessed for test-retest reliability. Second, the modest sample size and the non-probability nature of the sample may reduce generalizability of findings. While 62 observations were collected to assess test-retest reliability, participants belonged to four neighborhood quadrants pre-stratified based on income and walkability. This points to the need for care in extrapolating the reliability results beyond the study area and the need for reliability studies to be repeated when an audit instrument is used in other urban environments. A larger number of neighborhood quadrants would have allowed more precise estimation of reliability. Therefore, the test-retest reliability of the NEWS-India instrument should be investigated further across India.

A notable strength of this study was the recruitment of participants from four distinct neighborhood types, enhancing heterogeneity in socio-demographics, BE characteristics, and neighborhood walkability [134]. Another strength was the systematic adaptation of the NEWS, retaining most of the original items to allow for international comparison while tailoring the measure to reflect India-specific conditions. NEWS-India was developed through regional and international collaborations similar to those used for the NEWS questionnaire that was tailored to other LMICs such as Hong Kong [116], China [71], Brazil [74], Africa [65], and it was based on empirical evidence and test-retest reliability analysis with existing NEWS measures [65,70,74,77]. The present study supports the feasibility of using NEWS-India for assessing BE correlates of PA in India and can provide leads and guidance to researchers and practitioners in other Asian LMICs when evaluating the BE for health behaviors.

Overall, results indicate that NEWS-India was generally a reliable and practical instrument for collecting data and that participants found it easy to use. Findings such as these have international implications for utility and robustness of NEWS measures. This is important for identifying promising BE variables that could be policy targets or improving PA and controlling obesity worldwide. Use of adapted versions of the NEWS may be particularly important for research in LMICs that do not have geographic information systems databases or advanced technologies (e.g., global positioning systems or GPS, accelerometer data) that would allow objective measures of BE attributes and PA. While this instrument provides a method of collecting environmental data, it remains important to explore BE attributes that are key correlates of PA and whether these relationships are consistent across demographic groups, settings, and locations across India.

A major goal of IPEN is to represent the worldwide variation in BE, and conducting this study in an understudied LMIC like India adds to the international literature on the relevance of BE for promoting PA. The present study was the first to report on the psychometric properties of the NEWS in India, and suggests the need to create a version of NEWS that can be used within different populations and settings across India.

## 6. Conclusions

This study showed that NEWS-India has acceptable evidence of reliability. The development of culturally applicable NEWS measures for India is a necessary first step for supporting evidence-based interventions against the epidemics of inactivity-related NCDs. This study is first-generation research in India and has the potential to guide understanding of BE correlates of PA. Further adaptation, evaluation, and application of NEWS-India in other Indian states could lead to evidence-based recommendations for creating communities that are designed to make people more comfortable being physically active in India. Subscales of NEWS-India are related to constructs used in urban planning and transportation, findings based on these subscales can inform policies and interventions that may improve walkability and PA friendliness of a neighborhood. This is particularly important for physical activity and health promotion in India because chronic disease rates are rising in the region [7,9,135]. Understanding neighborhood BE correlates of PA is a priority that could lead to better strategies to prevent further declines in PA and promote PA among those physically inactive.

## Figures and Tables

**Table 1:** Themes and sample quotes (*N* = 21) across the socio-ecological framework related to the built environment and physical activity in Chennai, India, that informed the adaptation of NEWS-India.

Socio-Ecological Levels	Themes and Quotes
MICRO (e.g., individual, family, peers)	**INDIVIDUAL AND FAMILY FACTORS**
	*“There’s no space to walk on the sidewalks where you can walk carefully so that puts me off and I don't walk at all.” (R2)*
	*“It’s just not safe to walk outside, and it has become so bad that my mom said I want a treadmill at home and we bought a treadmill and my mom is walking on that.” (R5)*
	*“We did consider the children's school was nearby and my husband's office was nearby so that was a reason why we considered this (residential neighborhood).” (R2)*
	*“My parents are extremely spiritual people and there are lots of temples around, so they chose this house. And my dad he is diabetic, so walking is compulsory for him. So the beach is close by and that is why he liked this place.”*
MESO (e.g., connections between individual and the environment)	**NEIGHBORHOOD ENVIRONMENT**
	*“It is basically a concrete jungle.” (R1)*
	*“It (neighbourhood) used to be like a walk in a park, literally. In my neighbourhood before, there were open stretches of land, couple of residential houses, and no apartments. Now, there is hardly like any plot that is available for people to buy.” (R5)*
	*“Every household has at least one car and two bikes (motorized), and there is no place to place to park. There are many flats in Chennai that don’t have garages or parking facilities, so people end up parking on the roads. Those parked cars on either side make the roads even narrower to make things worse” (R5)*
	*“Green cover has come down by 50% since we moved in.” (R3)*
	*“Because of commercial activity, all the old houses are being pulled down and big complexes are coming up and all the trees are being cut down so all the shade is gone. As it is the weather is very hot and humid so it is not really favorable to walk in the hot sun in the afternoon.” (R2)*
	*“When it rains, we don’t have a proper sewage system, so the drainage and the sewage all comes out, the dirty water comes out on the roads and people have to travel. Because there are no sidewalks they end up traveling you know, through the muddy and dirty water.” (R5)*
MESO (e.g., connections between individual and the environment)	**PEDESTRIAN INFRASRUCTURE**
	*“There is a lot of unauthorized parking and the sidewalks have too many obstacles like some hawkers and laundry shops and garbage bins. And so it is like obstacle race if you want to walk on the sidewalk you have to keep getting up and down.” (R2)*
	*“I have aged during the last 22 years and have a problem with my knees. I just can't keep getting to such a high sidewalk again and getting down again.” (R2)*
	*There are many parts in the city where you will feel unsafe because streets are not lit up properly.” (R10)*
	**TRAFFIC SAFETY**
	*“Traffic has increased a lot on the roads and the traffic is not very pedestrian friendly and it's very difficult to walk. And to cross the road you have to like risk your life and cross the road.” (R2)*
	*“Motorists and the two-wheeler riders they don't respect it (crosswalks) at all so you have to be very, very careful while crossing the road.” (R2)*
	*“The traffic is chaotic and nobody respects the traffic rules. The motorists they jump signals. Even if it is a one-way street you see people coming the opposite direction. They just don't respect the traffic rules.” (R2)*
	*A lot of accidents are happening particularly on this road. I have at least seen some 3–4. In a day we at least have 4 ambulances coming and picking up people on this particular road.” (R7)*
	*“I have travelled kilometres to reach my home when it was raining one day because the traffic just would not budge, so we just got off whichever transport we were using, we walked like 10–15 km to reach my house.” (R1)*
	*“The roads were built for the number of people that were there say 50 years ago and we are still using the same (roads), and the population has multiplied by say a 100 or 1000 times.” (R5)*
	*“I make it a point not to step out of house because of the traffic and dirty roads. I feel I am safe at home than going out.” (R8)*
	*“Crossing the road, I feel sorry for senior citizens and the elderly.” (R10)*
	**PUBLIC TRANSPORT**
	*“Commuting there is pretty bad with regard to the roads and things especially public transport, there is hardly any transport there. There are no buses at all. Whatever it is, is like a few autos maybe 1 or 2 that commute to that area.” (R1)*
EXO (e.g., social networks and neighborhood community contexts, local politics and industry, mass media, social services)	**NEIGHBORHOOD COMMUNITY**
	*“One of the reasons they (parents) bought it (the house) was a sense of community. All the people around you belonged to the same community.” (R6)*
	*“This used to be a friendly, clean, and quiet neighbourhood. Before we used to walk on the road, stand at least near the gate and wave at each other. Now we don’t have any communication with our neighbours. It is so damn dirty and stinky, you don’t want to stand outside, so in that process we are losing our identity.” (R8)*
MACRO (e.g., attitudes and ideologies of culture, customs, and laws)	**POLICY**
	*“The road safety policy has never addressed pedestrian necessities.” (KI1)*
	*“Most of our roads are built only for cars, that’s the only demand that is visible for them (government), so many people have been coming up and saying you know, look at pedestrian safety.” (KI2)*
	**PLANNING**
	*“Fifty-two percent of city trips are less than 5 km, if you improve pedestrian infrastructure and improve cycle tracks, imagine how much fuel we would save.” (KI1)*
	*“They (city government) wanted a dedicated lane for pedestrians and cyclists, but somehow that plan took a back seat and now they want to build a 50 km elevated expressway again.” (KI2)*
	*“In many ways Indian traffic situation is really really bad, not only because it is overly congested compared to any other city around the world but it is also multi-vehicle, from bikes to say, any concept you try to apply, there is always a loophole. For example, it is very difficult to simulate these things anymore because behaviour of a bike or ‘thella gaadi’ (hand-drawn cart for transporting goods) and so on, is very different from the behaviour of a car.” (KI5)*
	**CITY-COMMUNITY PARTNERSHIP**
	*“The government has to work hand-in-hand with people.” (R5)*
	*They (local community) are not supportive about this whole widening of the pavement. Some of them, they say “Oh, the roads are already choked and by increasing the pavements you are going to choke it further, so where would our vehicles go?” (KI1)*
	**LAW ENFORCEMENT**
	*“Enforcements have to step up, traffic police is grossly understaffed.” (KI2)*
	*“Police only concentrate on getting the money out of the people, they are not bothered about regulating the traffic or doing something for the people.” (R12)*
	*“They (police) can put a firm hand and first of all on all these unauthorized shops and all the encroachments that have happened on the sidewalks. They have to be removed. Plus the unauthorized parking. I mean, you even find sometimes two-wheelers are parked on the sidewalks. So they have to take care of all these issues and like come down on all these people so that things improve for the general public as such.”*

Notes: R = Resident; KI = Key Informant.

**Table 2:** NEWS-India: answer frequencies, mean scores, and test-retest reliability scores for Residential Density.

Item/Scale	Answer Frequencies and Mean Score of Items on First Assessment (%) (*N* = 370)						Test-Retest Reliability Scores (*N* = 62)	
	None	A Few	Some	Most	All	Baseline Mean (SD)	Retest Mean (SD)	ICC
**Residential Density**								**0.88**
(a) independent houses or bungalow ^	21.4	13.5	14.3	28.4	22.4	3.17 (1.47)	3.63 (1.09)	0.91
(b) 1–3 storey flats or apartment buildings ^	15.4	15.1	20.0	34.1	15.4	3.19 (1.30)	3.79 (1.04)	0.88
(c) 4–6 storey flats or apartment buildings ^	49.7	10.0	10.8	19.5	10.0	2.30 (1.48)	2.79 (1.57)	0.96
(d) 7–12 storey flats or apartment buildings ^	86.2	5.9	4.6	1.4	1.9	1.27 (0.77)	1.21 (0.83)	0.83
(e) 13–20 storey flats or apartment buildings ^	93.5	3.0	1.6	0.5	1.4	1.13 (0.59)	1.10 (0.56)	0.95
(f) over 20 storey flats or apartment buildings *	96.5	1.9	0.8	0.0	0.5	1.05 (0.37)	1.03 (0.18)	1.00
(g) presence of slums *	51.6	14.6	8.4	6.2	18.9	2.25 (1.58)	2.42 (1.50)	0.88

Notes: ICC = Intra Class Coefficient; * Specific Indian items added to NEWS; ^ NEWS items with modified wording for Indian context.

**Table 3:** NEWS-India: answer frequencies, mean scores, and test-retest reliability scores for Land Use Mix-Diversity.

Item/Scale	Answer Frequencies and Mean Score of Items on First Assessment (%) (*N* = 370)						Test-Retest Reliability Scores (*N* = 62)	
	1–5 min	6–10 min	11–20 min	21–30 min	≥31 min	Baseline Mean (SD)	Retest Mean (SD)	ICC
**Distance to facilities (Land Use Mix-Diversity)**								**0.96**
(a) Provision store ^	3.5	4.6	11.4	25.9	54.6	4.24 (1.05)	4.53 (0.65)	0.78
(b) Supermarket	24.3	7.6	10.8	28.4	28.9	3.30 (1.55)	3.82 (1.34)	0.97
(c) Government ration shop *	27.6	11.9	15.7	25.7	19.2	2.97 (1.50)	1.97 (1.31)	0.93
(d) Milk booth *	21.1	3.5	10.3	26.5	38.6	3.58 (1.54)	4.26 (1.06)	0.95
(e) Fruit or vegetable market	21.6	7.6	10.8	29.2	30.8	3.40 (1.52)	4.02 (1.24)	0.95
(f) Meat or fish market *	19.2	12.2	22.2	29.7	16.8	3.13 (1.36)	3.35 (1.32)	0.95
(g) Street food vendors/food stalls *	20.0	9.5	14.1	34.9	21.6	3.29 (1.43)	3.56 (1.29)	0.95
(h) Food canteen *	50.3	15.7	14.6	13.8	5.7	2.09 (1.31)	1.89 (1.15)	0.97
(i) Fast-food restaurant	27.0	13.2	11.1	36.8	11.9	2.93 (1.43)	3.27 (1.39)	0.99
(j) Coffee shop ^	15.1	4.9	11.4	46.8	21.9	3.55 (1.30)	3.52 (1.25)	0.90
(k) Non-fast food restaurant	33.8	16.2	14.6	25.7	9.7	2.61 (1.42)	2.52 (1.43)	0.92
(l) Street vendors *	40.5	15.1	16.8	20.5	7.0	2.38 (1.37)	2.39 (1.25)	0.93
(m) Shops and stores ^	28.6	17.0	20.8	24.3	9.2	2.68 (1.35)	2.52 (1.35)	0.94
(n) Hardware or building material store ^	25.7	14.3	20.8	32.7	6.5	2.80 (1.31)	1.84 (1.28)	0.86
(o) Telephone booth *	24.3	3.0	6.8	39.5	26.5	3.41 (1.52)	3.81 (1.16)	0.77
(p) Printing/Xerox shop *	14.9	7.8	9.2	41.6	26.5	3.57 (1.35)	3.94 (0.96)	0.81
(q) Dry cleaner/ironing ^	17.6	5.4	8.4	38.1	30.5	3.59 (1.42)	4.05 (1.02)	0.89
(r) Tailor, cobbler *	19.7	14.1	15.1	33.2	17.8	3.15 (1.40)	3.15 (1.46)	0.90
(s) Post office	41.1	11.4	17.0	25.1	5.4	2.42 (1.38)	2.56 (1.36)	0.95
(t) Library	53.8	12.4	16.8	15.7	1.4	1.98 (1.21)	2.31 (1.26)	0.91
(u) School	20.8	10.0	21.1	39.7	8.4	3.05 (1.29)	3.32 (1.16)	0.84
(v) College or university ^	62.2	19.2	10.3	6.2	2.2	1.67 (1.03)	1.84 (1.09)	0.97
(w) Book store	39.7	14.6	17.6	25.9	2.2	2.36 (1.30)	2.60 (1.29)	0.92
(x) Bank or cooperative bank ^	28.9	9.5	11.4	45.1	5.1	2.88 (1.38)	3.45 (1.04)	0.93
(y) Shopping mall *	65.9	14.1	10.8	6.5	2.7	1.66 (1.08)	1.66 (1.07)	0.99
(z) Movie theater or multiplex *	69.2	14.9	8.4	5.1	2.4	1.57 (1.01)	1.52 (0.92)	0.97
(aa) Video/music CD store^	56.2	16.5	15.7	9.2	2.4	1.85 (1.13)	1.73 (1.09)	0.95
(bb) Pharmacy or medicine shop ^	11.9	10.5	13.2	54.1	10.3	3.40 (1.17)	3.77 (0.88)	0.81
(cc) Salon ^	23.2	13.2	12.4	42.2	8.9	3.00 (1.36)	3.19 (1.19)	0.79
(dd) Your job or school	61.4	8.9	7.0	19.7	3.0	1.94 (1.32)	2.21 (1.45)	0.93
(ee) Bus stop or railway station	20.3	6.5	13.0	45.4	14.9	3.28 (1.36)	3.76 (1.10)	0.88
(ff) Taxi or auto rickshaw stand *	14.1	7.3	12.7	49.7	16.2	3.47 (1.25)	3.79 (1.07)	0.90
(gg) Mechanic or repair shop *	16.8	14.9	24.3	34.3	9.7	3.05 (1.25)	3.32 (1.05)	0.85
(hh) Park or green space ^	42.7	7.0	14.6	27.0	8.6	2.52 (1.47)	2.66 (1.49)	0.99
(ii) Playground *	42.4	7.6	14.1	28.9	7.0	2.51 (1.45)	2.94 (1.45)	0.96
(jj) Open field/school field *	39.2	10.5	17.6	26.5	6.2	2.50 (1.39)	2.92 (1.37)	0.94
(kk) Club or recreation center ^	48.9	4.9	12.4	29.7	4.1	2.35 (1.43)	2.95 (1.31)	0.95
(ll) Gym or fitness facility	35.4	8.9	15.9	33.2	6.5	2.66 (1.41)	3.24 (1.22)	0.98
(mm) Private clinic/hospital *	23.8	16.8	16.8	37.6	5.1	2.84 (1.30)	3.31 (1.08)	0.97
(nn) Government hospital *	42.2	15.1	13.2	28.1	1.4	2.31 (1.31)	2.61 (1.21)	.94
(oo) Tap, well/common water source *	68.9	8.4	4.1	12.2	6.5	1.79 (1.33)	1.71 (1.31)	0.90
(pp) Place of worship *	14.6	12.2	18.6	45.9	8.6	3.22 (1.21)	3.50 (0.92)	0.91
(qq) Beach *	81.9	10.0	1.9	3.0	3.2	1.36 (0.92)	1.42 (0.97)	0.96

Notes: ICC = Intra Class Coefficient; * Specific Indian items added to NEWS; ^ NEWS items with modified wording for Indian context.

**Table 4:** NEWS-India: answer frequencies, mean scores, and test-retest reliability scores for Land Use Mix-Access, Street Connectivity, and Infrastructure and Safety for Walking/Bicycling.

Item/Scale	Answer Frequencies and Mean Score of Items on First Assessment (%) (*N* = 370)					Test-Retest Reliability Scores (*N* = 62)	
	Strongly Disagree	Somewhat Disagree	Somewhat Agree	Strongly Agree	Baseline Mean (SD)	Retest Mean (SD)	ICC
**Land Use Mix-Access**							**0.98**
(a) possible to do shopping at local stores	13.2	11.1	28.1	47.6	3.10 (1.05)	3.39 (0.88)	0.95
(b) shops within easy walking distance	11.4	5.7	27.6	55.4	3.27 (0.99)	3.40 (0.84)	0.97
(c) many places in walking distance of home	15.9	10.5	25.9	47.6	3.05 (1.10)	3.10 (1.13)	0.99
(d) easy to walk to transit stop	13.5	6.8	31.6	48.1	3.14 (1.04)	3.29 (0.89)	0.91
**Street Connectivity**							**0.85**
(a) distance between road junctions is short	18.1	10.3	38.1	33.5	2.87 (1.07)	3.45 (0.72)	0.83
(b) many four-way road junctions	13.8	8.6	40.3	37.3	3.01 (1.01)	3.45 (0.74)	0.79
(c) many alternative routes	10.8	7.3	50.0	31.9	3.03 (0.91)	3.11 (0.73)	0.89
**Infrastructure and Safety for Walking/Bicycling**							**0.97**
(a) footpaths/pavements on most streets ^	43.8	10.3	19.2	26.8	2.29 (1.27)	2.48 (1.36)	1.00
(b) footpaths/pavements well-maintained ^	58.8	10.8	17.3	13.0	1.85 (1.12)	1.48 (0.95)	0.95
(c) bicycle or pedestrian pathways are easy to get to	75.9	10.8	10.0	3.2	1.41 (0.80)	1.19 (0.54)	1.00
(d) footpaths separated from road by parked cars, motorcycles, or auto-rickshaws ^	56.2	9.7	25.1	8.9	1.87 (1.08)	2.08 (1.06)	0.70
(e) footpaths separated by grass/dirt strip	74.6	12.4	7.6	5.4	1.44 (0.85)	1.13 (0.38)	1.00
(f) safe to ride a bicycle	64.6	8.6	17.8	8.9	1.71 (1.05)	1.42 (0.82)	0.93
(g) streets well-lit at night	17.6	6.8	28.1	47.6	3.06 (1.12)	3.21 (1.04)	0.96
(h) walkers and bicyclists easily seen by people in homes	16.2	9.5	54.1	20.3	2.78 (0.95)	2.97 (0.72)	0.88
(i) zebra crossings present ^	66.2	7.0	17.0	9.7	1.70 (1.07)	1.39 (0.86)	0.98
(j) zebra crossings promote safety ^	64.9	7.3	17.8	10.0	1.73 (1.08)	1.37 (0.87)	0.93
(k) footpaths not obstructed *	60.3	11.6	13.5	14.6	1.82 (1.14)	1.48 (1.02)	0.99
(l) facilities to bicycle (lanes, *etc.*) available *	80.0	8.4	8.4	3.2	1.35 (0.77)	1.11 (0.45)	0.48

Notes: ICC = Intra Class Coefficient; * Specific Indian items added to NEW; ^ NEWS items with modified wording for Indian context.

**Table 5:** NEWS-India: answer frequencies, mean scores, and test-retest reliability scores for Safety from Traffic and Safety from Crime.

Item/Scale	Answer Frequencies and Mean Score of Items on First Assessment (%) (*N* = 370)					Test-Retest Reliability Scores (*N* = 62)	
	Strongly Disagree	Somewhat Disagree	Somewhat Agree	Strongly Agree	Baseline Mean (SD)	Retest Mean (SD)	ICC
**Aesthetics**							**0.93**
(a) presence of trees	20.5	11.9	36.5	31.1	2.78 (1.10)	2.81 (1.13)	0.91
(b) trees give shade	25.4	12.2	36.5	25.9	2.63 (1.12)	2.63 (1.18)	0.94
(c) interesting things to look at	49.7	20.3	24.3	5.7	1.86 (0.98)	1.48 (0.81)	0.90
(d) neighborhood is free from litter/garbage, graffiti, or stagnant water *	66.8	14.3	10.0	8.9	1.61 (0.99)	1.37 (0.81)	0.96
(e) attractive natural sights	62.7	19.2	13.5	4.6	1.60 (0.89)	1.37 (0.71)	0.78
(f) attractive buildings	56.2	18.4	19.2	6.2	1.75 (0.97)	1.27 (0.58)	0.79
**Safety from Traffic**							**0.90**
(a) traffic on street I live makes it difficult to walk	25.5	14.4	20.3	39.8	2.75 (1.23)	2.95 (1.15)	0.95
(b) traffic on nearby streets makes it difficult to walk	21.4	10.8	21.1	46.6	2.93 (1.20)	3.53 (0.82)	0.71
(c) speed of traffic on street I live is slow	30.1	17.9	34.1	17.9	2.40 (1.10)	2.37 (1.01)	0.85
(d) speed of traffic on nearby streets is slow	36.3	21.1	28.2	14.4	2.21 (1.09)	1.98 (1.00)	0.81
(e) drivers exceed speed limits	13.6	18.7	29.0	38.8	2.93 (1.06)	3.11 (0.96)	0.87
(f) a lot of exhaust fumes	16.3	12.7	28.2	42.8	2.98 (1.10)	3.15 (1.13)	0.93
**Safety from Crime**							**0.92**
(a) high crime rate	29.7	12.2	28.4	29.7	2.58 (1.20)	2.63 (1.24)	0.89
(b) unsafe to walk during day	33.0	23.5	21.2	22.4	2.33 (1.15)	1.98 (1.06)	0.84
(c) unsafe to walk at night	25.5	16.0	25.5	32.9	2.66 (1.18)	3.02 (1.20)	0.91
(d) neighborhood safe for a 10-year old boy to walk alone in daytime	19.5	13.2	42.4	24.9	2.73 (1.04)	2.73 (0.91)	0.85

Notes: ICC = Intra Class Coefficient; * Specific Indian items added to NEWS.

**Table 6:** NEWS-India: answer frequencies, mean scores, and test-retest reliability scores for Single Items.

Item/Scale	Answer Frequencies and Mean Score of Items on First Assessment (%) (*N* = 370)					Test-Retest Reliability Scores (*N* = 62)	
	Strongly Disagree	Somewhat Disagree	Somewhat Agree	Strongly Agree	Baseline Mean (SD)	Retest Mean (SD)	ICC
**Single Items**							
(a) parking is difficult in local shopping areas	23.8	11.6	20.5	44.1	2.85 (1.22)	3.03 (1.20)	0.97
(b) streets are hilly	80.3	7.0	8.6	4.1	1.36 (0.81)	1.06 (0.25)	1.00
(c) major barriers to walking (bad roads, poor sidewalks, poor drainage, water logging) ^	21.6	11.4	15.7	51.4	2.97 (1.22)	3.63 (0.79)	0.81
(d) streets do not have many dead-ends	31.6	27.6	28.4	12.4	2.22 (1.03)	2.03 (0.89)	0.70
(e) walking paths connect dead-ends to main roads or streets ^	37.0	13.0	37.3	12.7	2.26 (1.09)	2.73 (0.83)	0.50
(f) see and speak to other people while walking in neighborhood	16.2	5.4	52.7	25.7	2.88 (0.97)	2.89 (0.85)	0.85

Notes: ICC = Intra Class Coefficient; ^ NEWS items with modified wording for Indian context. Single item subscales used in this study were based on those suggested by a multi-level confirmatory factor analysis of the original NEWS scoring [66].
